# Some Further Thoughts Concerning the Origin and Spread of Yellow Fever, and the Means of Preventing It

**Published:** 1879-12

**Authors:** N. S. Davis

**Affiliations:** Chicago, Ill.


### Original Communications.

ARTICLE II.

### SOME FURTHER THOUGHTS CONCERNING THE ORIGIN AND SPREAD OF YELLOW FEVER AND THE MEANS OF PREVENTING IT. By N. S. DAVIS, M.D., Chicago, Ill. Read to the Evanston Philosophical Society.

During the progress of the severe epidemic of yellow fever in the summer of 1878, in the lower part of the valley of the Mississippi, I wrote an article on this subject, which was read to this society and published in the *Maryland Medical Journal* for October, 1878. In that article I endeavored to show that while we were not able, in the present state of medical knowledge, to point out the special specific cause, *materies morbi* (if such exists), which causes the disease called yellow fever, it was possible to state several of the conditions necessary for its production and propagation; and further, that at least some of these conditions are amenable to human control. I ventured also to specify some of the means for gaining such control over the production of the cause, and for limiting its spread or propagation after it had been produced. And I come now to inquire whether the re-appearance of the disease in some places on the Mississippi the past summer, and the investigations of the past year, have developed any new facts or thrown any new light upon either the origin or the means of prevention of the disease. If the questions involved in a consideration of this subject were of interest to physicians and their patients only, I would not occupy your time with it. But you are well aware that no questions bear a more direct relation to the social, industrial and commercial interests of the country than those relating to the origin and spread of severe epidemic diseases. Such diseases cannot prevail in any part of our country without causing such destruction of life, suspensions of industry and interruptions of commerce as to be felt throughout the whole land. Therefore, whatever relates to their causes, modes of propagation and means of prevention, is of interest to all classes of the community. In my former paper, to which allusion has been made, I stated the following propositions as fully sustained by the history of all previous epidemics:

"1st. The essential cause of the disease, whether animal, vegetable, or chemical, depends for its development and propagation or spread, on conditions of a strictly local character, found co-existing only within certain geographical limits.

"2d. The most important of the necessary co-existing conditions so far as ascertained, are, continuous high temperature, excess of atmospheric moisture, the presence in the atmosphere of the products of animal or vegetable decomposition, and such geographical position in relation to latitude and altitude as to secure a great predominance of the warm over the cold season of the year. The absence of any one of the first three conditions here named, positively prevents the development or spread of the disease. On that part of the northwestern coast of Africa bordering on the Atlantic and the Mediterranian, and the West India Islands where these conditions always co-exist in a prominent degree, the disease is indigenous or endemic, prevailing more or less during the warm season of almost every year; while within certain limits, both north and south of the regions just named, the cause of the disease may be either developed locally or imported from other places and spread, whenever a season occurs in which the essential conditions co-exist in an unusual degree.

"3d. As all the known conditions essential to the development and spread of the disease, pertain to the atmosphere, it is plain that the essential cause must be developed in the atmosphere and not in the living human body. Consequently if such cause spreads or becomes transferable from one place to another, it is solely by atmospheric infection and not from personal contagion. And no amount of atmospheric infection by importation or otherwise can propagate the disease or its causes, in any locality where the atmosphere does not contain the required temperature and local impurities.

"4th. The causes and conditions giving rise to the disease being exclusively of local atmospheric origin, are neither developed in nor evolved from the bodies of the sick; and, hence, the transference of any number of persons, whether sick or well, from infected places to localities where the essential atmospheric conditions do not exist, never has propagated the disease in the latter places and never can."

It will be perceived that the words atmospheric *infection* and personal *contagion* are used in the foregoing propositions, and it may prevent confusion of thought or misunderstanding, if I explain that the former relates to a poison generated outside and independent of the human body, and its propagation is wholly dependent on certain atmospheric conditions; while the latter is always generated in the living body and communicable from one individual to another by contact or near proximity without any regard to the conditions of the surrounding atmosphere. Hence the diseases produced by the infections prevail only within certain climatic and geographical limits and at certain seasons of the year, of which intermittent, remittent, and yellow fevers, and cholera are noted examples; while those arising from personal contagion prevail in any locality or climate and at any season of the year, of which small-pox, measles, scarlatina, and whooping-cough, are familiar examples.

In my former communication many facts were given from the history of former epidemics in this and other countries, sustaining more or less fully, the truth of the four propositions just stated.

Instead of repeating these, I shall simply inquire how far the facts developed in connection with the epidemics of the two past summers (1878 and 1879), sustain or disprove these same propositions. The most important items in these several propositions are contained in the first paragraph of the second general proposition, which asserts that the conditions essential for the origination and spread of yellow fever, are the co-existence of continuous high temperature, excess of atmospheric moisture, the presence in the atmosphere of the products of animal or vegetable decomposition, and such geographical position in regard to latitude and altitude as to secure a decided predominance of the warm over the cold season of the year.

During the last few months, Dr. W. Hutson Ford of St. Louis, has published the results of observations concerning the relations of temperature to the prevalence of yellow fever. He has been enabled to compare the meteorological and mortuary records of Charleston, South Carolina, through a period of thirty-eight years, and of ten other southern cities for a period of five years. Of the thirty-eight years included in the records in Charleston, seventeen were characterized by more or less prevalence of yellow fever. In six of these eighteen years the disease assumed a severe epidemic form; in six, mildly epidemic, and in five, only a few sporadic cases occurred. Only twice during the whole period did the disease prevail in decided or severe epidemic form two years in succession. In nearly all instances only sporadic or scattering cases occurred the summer succeeding a severe epidemic. The commencement of the disease was generally in August and its prevalence was limited to the months of August, September and October. On comparing the meteorological with the mortuary records for the whole period of thirty-eight years, Dr. Ford found that the years in which the yellow fever was epidemic were the same in which the summer heat rose to the highest *mean* for the three months just named in each year. The six years of severe epidemic prevalence were also the six years giving the maximum mean temperature of the summer months. The six years of slight epidemics ranked next in the mean temperature of the same months.

The five years of sporadic cases gave a mean temperature for summer and autumn less than those in which the disease was moderately epidemic. In the remaining twenty years in which there was no prevalence of the yellow fever, the mean temperature of the summer months was at the minimum; the highest of any of these years being lower than the lowest of those in which the disease prevailed. The only exception to this rule was in 1836, when the mean temperature of the months of July, August, September and October, was as high as the years of most severe epidemic prevalence of yellow fever, and in that year the city was scourged by a severe epidemic of cholera, that appeared to supercede the yellow fever. Dr. Ford has analysed and compared these statistics of Charleston in the most varied and philosophical manner, but always arriving at the same result, namely, that the seasons of yellow fever epidemics are identical with those of highest summer temperature. His comparison of meteorological and mortuary statistics in the other ten cities situated on the Gulf of Mexico and along the Mississippi river, as far north as St. Louis and Louisville, is only for a period of five years including 1874-5-6-7-8. But they lead to precisely the same conclusions.

Thus the summers of 1873-4 were a little above the average mean for a series of ten years, and there were moderate epidemics of yellow fever in several of the cities on the lower Mississippi and the Gulf. The summers of 1875-6-7 were decidedly below the mean temperature for a series of ten consecutive years, and there were no epidemics of the fever in any of the cities under consideration. The mean temperature for July, August, September and October, was the lowest in 1875, from which an annual increase was presented in 1876 and 1877, culminating in the extraordinary summer temperature of 1878, and the equally extraordinary epidemic prevalence of the the disease. The mean temperature of the summer of 1879, just closed, falls below that of 1878, yet is decidedly above the average for a series of years, especially in the middle and lower part of the Mississippi valley. And true to the law, already deduced, the yellow fever re-appeared fairly epidemic in Memphis and its vicinity, and sporadically in New Orleans and a few other places. These eminently philosophical deductions of Dr. Ford, are corroborated by a great variety of other facts; and are sufficient to show a *necessary* connection between unusual high summer temperature and the appearance of yellow fever epidemics. If the investigations related only to the years 1878 and 1879, or to any other one or two years, the co-existence of a high mean summer temperature and an epidemic prevalence of the fever might be regarded as merely accidental; but when the statistics cover a period of thirty or forty consecutive years, as in the case of Charleston, and the same co-existence is found uniform throughout, the presumption of accidental coincidence ceases, and the deduction assumes the importance of a fixed law. The same series of investigations and statistical comparisons also establish the important fact that the fever never assumes an epidemic character until the high summer temperature has progressed two months, namely, through the months of June and July; the favorite month for its epidemic ravages to commence in our country being August. And in the few instances of its commencing to prevail epidemically in July, it is found that the high summer heat had commenced in May. Such was the case in Memphis the past summer. In the temperate zone the sun reaches a position relative to the earth which gives to its rays most directness and power to impart the greatest amount of heat to the earth's surface, about the 21st of June. At the same time, the days become the longest compared with the nights, and consequently, more heat is absorbed each day by the earth than is radiated into the air during the night; and hence there is a steadily increasing temperature of the earth's surface through June, July and August, while that of the atmosphere may be much more fluctuating. Often times, even here in Chicago, the mercury rises higher for a few hours in the middle of some days in the third week in June, than in any other days of the year, but the nights are yet cool, and no visible disturbances of health result from such temporary high temperature. So also many instances have occurred where ships having yellow fever on board have arrived in New Orleans and other Gulf or Atlantic ports, during the months of May and June, and even here and there a sporadic case has occurred in those cities independent of any known importation, during those months, yet no general or epidemic development has appeared, until the latter part of July or in August, and in many instances, not until early in September. These facts show that it is not merely high temperature, but such temperature continued until the surface of the earth reaches a degree of heat and moisture most favorable for rapid decomposition of organic matter, and the suspension of the products of such decomposition with aqueous vapor in the atmosphere, that we get the exact meteorological condition *necessary* for originating or sustaining an epidemic of yellow fever. But even when the temperature is sufficiently high and long continued, with a moisture most favorable for fermentation or decomposition of organic matter, yet no epidemic yellow fever will be developed unless the particular kind of organic matter required be present to undergo such change, in sufficient quantity to impregnate the atmosphere to a considerable extent. Just what the deleterious material is, that is engendered and diffused in the atmosphere as the pabulum for supporting yellow fever, is not yet known. Neither is it fully known as to what kind of fermentive or decomposible organic matter is necessary to furnish the pabulum on which the heat and moisture is to act. Yet all the facts connected with the origin and progress of the yellow fever during the last two summers, point unmistakably to local atmospheric and topographical conditions as exerting a controlling influence over the spread or continuance of the disease, whether its supposed essential cause was imported or not. For instance, in the summer of 1878, the disease assumed an epidemic form in New Orleans during the month of July, and prevailed nearly a month before any cases were recognized in Memphis, although there was constant communication between the two cities both by river and railroad. And at a still later period the disease made its appearance in many smaller places, more or less distant from each other, so nearly simultaneously as to preclude the possibility that it had been communicated from one to another.

Again, in the present year, as is well known, the disease commenced in Memphis, and prevailed severely before it had appeared in New Orleans, or any other place bordering on the Gulf. More than half the population speedily abandoned the city, scattering themselves widely over the more northern parts of the country; many hundred more went into camps on well chosen ground only twenty or thirty miles distant from the city; while the local board of health, aided by the State and national health organizations, not only established and enforced the most rigid quarantine, but waged an unceasing warfare upon the disease in the city by isolation and disinfection, using almost unlimited quantities of the best antiseptic and disinfecting remedies known; and yet the epidemic continued the even tenor of its way in the city, and after five weeks made its appearance in a score or more of smaller places in different directions from Memphis. But the moment a severe frost made its appearance, reducing the temperature of the atmosphere a little below 0° C., or 32° F., the disease, which for nearly three months had bid defiance to hundreds of tons of disinfectants and any number of quarantines, even when aided by shot-guns, disappeared as if by magic. If we put the facts recently developed in regard to the necessary influence of continuous high temperature in originating an epidemic of yellow fever, with the long known fact that a *low* temperature invariably extinguishes it, we have proof amounting to demonstration that the propositions already stated in relation to the several conditions that *must co-exist* in any given locality to allow the development of an epidemic of this disease, are correct.

In relation to the most efficient means of preventing the development and spread of yellow fever, I stated in my former communication, "that the first and most important of all the measures for preventing the development of yellow fever in any given place, either locally or by importation, are such as will remove one or more of the conditions necessary for the production or spread of the disease. Of these conditions, the one most readily under human control is the contamination of the atmosphere from local sources of vegetable and animal decomposition. It is well known that the chief sources of such decomposition are imperfect and uncleanly sewers or cess-pools, foul and stagnant water, and low, moist ground, rich in vegetable matter.

"To remove these sources of atmospheric impurity early in each year, and keep them thoroughly removed until the close of the warm season, and thereby prevent the supply of local material on which the essential cause of yellow fever depends for its propagation, is the *only reliable* safeguard against the development of this disease in any place within the geographical range of its prevalence. If this is neglected until the atmosphere of any locality becomes filled with miasms as a pabulum for the fever poison, and the summer temperature prove continuously high, the disease will prevail and spread in defiance of all the inland quarantines that can be devised. But if a sufficient degree of cleanliness in regard to streets, alleys, gutters, sewers and stagnant waters, to prevent the atmosphere from becoming filled with the products of decomposition and impurities, is accomplished early in the season and faithfully maintained until the frosts of autumn, there will be no danger of the prevalence of yellow fever, either by importation or otherwise. The point of vital importance is to *prevent* the development of the noxious material that constitutes the pabulum on which the essential cause of the disease feeds or out of which it originates.

The second series of preventive measures of great importance is suggested by the third and fourth propositions stated above, and are as follows:

"1st. The municipal and health authorities of every important city or town on the coast of the Gulf, from the Mexican boundary to Charleston on the Atlantic; on the Mississippi from New Orleans to St. Louis; on the Red River below Shreveport; on the Ohio below Pittsburgh; and on the principal lines of railroad in immediate connection with such cities and towns, should deliberately select the nearest unoccupied, dry, elevated place, containing pure air and good water, and as readily accessible as possible, to which all families willing to go could be speedily removed from an infected street or section of a town or city, and accommodated in tents or other temporary structures until they could safely return to their homes.

"Wherever this principle of speedy removal was acted upon by our army, it proved entirely successful in stopping the spread of the disease among the soldiers, and its imperfect and limited adoption at Memphis the present season has been of great value.

"If the proper places were carefully selected beforehand, and a supply of tents or other material kept under the control of the proper authorities, so that on the first appearance of the disease in a neighborhood those exposed could be removed without delay, and ordinary supplies of provisions for the poor dealt out *only* at the camp or camps, there would be but little difficulty in limiting the local spread and fatality of any epidemic. While such camps would chiefly operate for the benefit of the poorer classes (and wherever it should be possible to find the proper grounds on railroad lines within a radius of from ten to twenty miles of the city, many of the workingmen could go in every morning and continue many kinds of work), all who were able to provide for themselves and their families away from home and were not thoroughly acclimated, should be encouraged to go early and freely, the only condition imposed being that they should not stop until they had passed entirely north of the climatic zone of the yellow fever. The only internal or inland quarantine regulations required are the selection of suitable and well prepared healthy stations a few miles from each of the more important cities on the great lines of travel, whether by river or railroad, where boats and trains shall halt long enough for inspection, and if any are found sick of the fever they shall be transferred directly to the station and cared for, the boat or car being thoroughly ventilated, and allowed to proceed with all the well persons, to any proper northern destination. The great northwestern region bounded on the east by Waukesha, Mackinac and Marquette, extending indefinitely westward over the northern peninsula of Michigan, northern Wisconsin and Minnesota; and the whole Alleghany range in the northeast, from Virginia to the Adirondacks, are sufficient to accommodate every unacclimated person in the lower Mississippi Valley and in our southern seaport cities; and they could no more spread the yellow fever in those regions than intermittent fever could be spread on Mount Washington.

"2d. The same principles apply to commerce and business. There is no positive evidence whatever, that the disease is ever transmitted by simple contact with the sick, nor by either articles of clothing or merchandise that have been freely exposed to the air outside of an infected locality. It is only when the *infected air* of the locality where the disease is prevailing, is shut up in the hold or apartments of a ship, boat or car, or boxed up with goods in boxes or trunks that it can be carried to distant places and retain its active properties. And even when so carried, it must be let out in an atmosphere in the new locality at the proper high temperature and containing the necessary local miasms or impurities, or it becomes utterly harmless. All that is necessary, therefore, is to have all ships, boats, and cars, carrying freight, stopped at suitable places outside of populous towns, inspected, all parts thoroughly ventilated and cleansed; and where goods had been packed in bales, boxes, or trunks, the same opened and aired before they are received by the parties to whom they are consigned. If the municipal and health officers throughout the Mississippi Valley had been prepared, at the first beginning of the present epidemic in New Orleans, to have acted promptly in accordance with the foregoing suggestions, and their action been cordially sanctioned by the people of the North, how vastly different would have been the result. Instead of throwing every possible obstacle in the way of the escape of the unacclimated and timid from the infected city or cities; stopping commerce and business, and consequently supplies; throwing laborers out of every kind of employment, thereby adding to the dread of pestilence, the still more destructive horrors of famine, idleness, and isolation from the rest of the world; tens of thousands of those who have been compelled to remain, substantially imprisoned within the infected regions, or to overcrowd the few places, like Nashville, Holly Springs, Louisville, and to a less degree, Cincinnati and St. Louis, near the borders of the climatic yellow fever zone, would have been enjoying health and vigor in the regions indicated in the north; other thousands of the poor would have been in healthy encampments, where their wants could have been easily and regularly supplied; the business of the country would have proceeded in its ordinary channels, and the lives of at least one-half of those who have fallen victims to the pestilence would have been saved."

It must be remembered that these suggestions in regard to preventive measures were written while the epidemic of 1878 was in its most active stage of progress. It is now a matter of interest to enquire whether anything has since transpired, calculated to either sustain or disprove their correctness. In regard to preventing the atmosphere from becoming impregnated with the products from decomposition of vegetable and animal matter, by efficient drainage, strict cleanliness, and the proper use of disinfectants all the year, but especially during the three months preceding the climax of summer heat, not enough has been done to afford a fair test of its value. The proposition that, on the appearance of an epidemic in any given place, all persons not being fully acclimated or protected by previous attacks of the disease, should be encouraged to immediately remove to healthy districts; such as were pecuniarily able to go beyond the yellowfever zone, and those who were not able, to go into well-selected camps in the vicinity, was subjected to a pretty fair test in Memphis the past summer.

On the outbreak of the epidemic the utmost facilities were afforded for all who wished to go to the North, and three camps were established in judiciously selected localities within twenty or thirty miles of the city, in which many hundreds of the poorer classes took refuge. The result was most gratifying. Those in the camps remained perfectly free from the disease; only a very few of those who fled to the North were taken sick after their departure, and yet the population left in the city was reduced to ten or twelve thousand, and the aggregate number of deaths from the fever during the whole season was only about 550, instead of 2,500 during the epidemic of 1878.

The suggestion to carefully select stations in proper places along the lines of travel and commerce, both by rivers and railroads, at which boats or cars from infected places should be stopped for inspection, and, when necessary, thoroughly ventilated and cleansed, with the removal of any found sick to a hospital for proper care, while the well were allowed to proceed on their way, therebysubstituting systematic inspection, with enforcement of ventilation, cleanliness, and care of the sick, in the place of quarantines, has been tested only to a limited extent. A station of this kind was established the past summer at Island No. 1, below Cairo, and in a less perfect manner on the Ohio, below Louisville and Cincinnati. The quarantine station, fourteen miles below St. Louis, was also managed partly on the same plan. The results at each of these places has been most beneficial, and fully demonstrates that if the plan of establishing *inspection stations*, with temporary hospital accommodations attached, was carried out in the systematic manner I have suggested, it would afford a far better protection against the spread of the disease from one place to another than the ordinary methods of quarantine, even if aided by shot-guns, and yet neither destroy commerce nor interrupt labor.

The question is often asked whether there is any danger of yellow fever in Chicago? We may confidently answer this question in the negative, if there is such necessary connection between continuous high temperature and the prevalence of the disease as indicated by the foregoing meteorological facts. So far as yet ascertained, the fever has not occurred in any place in the Southern States as an epidemic, however mild, until after at least two months of *mean* summer heat, ranging from 21.3° C. to 26.8° C., continuing at 26.8° C., or above, for August and September, and from 24.1° C. to 26.8° C. for October.

It is well known that we have no seasons of such protracted high heat here. For instance the temperature of the past summer (1879) was above the average for a series of years; yet the mean temperature for July was only 24.6° C.; that of August 22.7° C.; September, 16.1° C., and October, 16.6° C. It may be proper to remark that the mean temperature of the last named month was at least 2.7° C. above the usual average mean for October. But, while the temperature of our summers is too low to favor the prevalence of yellow fever, it would be a great mistake to suppose that it was not sufficient to produce a great effect on the prevalence and mortality of some other diseases. On the contrary, the sudden accession of continuous high heat in July, as compared with May and June, uniformly induces a rapid development of bowel affections, as diarrhœa, cholera morbus, and cholera infantum, which prove fatal to large numbers of children under three years of age. Indeed, a larger number of children under three years of age died in the city of Chicago during the months of July and August, 1879, than have died of yellow fever during the whole of the same year in all the Southern States. And the unusual high mean temperature of the past October was accompanied by 112 more deaths in this city than during the corresponding month in 1878, the mean temperature of which was about 5° C. lower. It is thus seen that meteorological conditions exert an important, if not controlling, influence over the prevalence of ordinary endemic, as well as over extraordinary epidemic diseases.

